# Temporal Association Rule Mining and Updating and Their Application to Blast Furnace in the Steel Industry

**DOI:** 10.1155/2020/7467213

**Published:** 2020-05-11

**Authors:** Yinghua Han, Deshui Yu, Chunhui Yin, Qiang Zhao

**Affiliations:** ^1^School of Computer and Communication Engineering, Northeastern University at Qinhuangdao, Qinhuangdao, China; ^2^School of Control Engineering, Northeastern University at Qinhuangdao, Qinhuangdao, China

## Abstract

Blast furnace (BF) is the main method of modern iron-making. Ensuring the stability of the BF conditions can effectively improve the quality and output of iron and steel. However, operations of BF depend on mainly human experience, which causes two problems: (1) human experience is not objective and is difficult to inherit and learn and (2) it is difficult to acquire knowledge that contains time information among multiple variables in BF. To address these problems, a data-driven method is proposed. In this article, we propose a novel and efficient algorithm for discovering underlying knowledge in the form of temporal association rules (TARs) in BF iron-making data. First, a new TAR mining framework is proposed for mining temporal frequent patterns. Then, a novel TAR mining algorithm is proposed for mining underlying, up-to-date, and effective knowledge in the form of TARs. Finally, considering the updating of the BF database, a rule updating method is proposed that is based on the algorithm that is proposed in this article. Our extensive experiments demonstrate the satisfactory performance of the proposed algorithm in discovering TARs in comparison with the state-of-the-art algorithms. Experiments on BF iron-making data have demonstrated the superior performance and practicability of the proposed method.

## 1. Introduction

Iron and steel are two of the most important raw materials in modern society. Their quality and output are not only important indices for measuring a country's economic strength but also play an incomparable role in a country's development. The iron-making process is the upstream process of the iron and steel industry; thus, it is important for the output and quality of the whole iron and steel production process. In the iron-making production, blast furnace (BF) iron-making has always occupied a dominant position, and its output accounted for more than 95% of the world's steel production.

Stable furnace conditions are the main prerequisite for high-quality steel production. However, the BF iron-making process is a typical complex nonlinear system, which contains hundreds of physical and chemical reactions [1, 2]. In addition, BF is a typical black-box system, and its smelting process has the following characteristics: multivariable coupling, large time delay, and nonlinearity. These characteristics increase the probability of abnormal conditions in BF, which will affect the quality and output of steel. These characteristics render it difficult to discover the temporal relationships among BF variables for BF operators and increase the difficulty of BF operation. Because of the complexity of BF smelting, it is difficult to construct an accurate model via the traditional mechanism model method; therefore, it is difficult to identify the temporal relationships among multiple variables to help stabilize the furnace conditions.

With the automation of BF and the development of the Internet of things (IoT), the production data of BF can be easily obtained and form a historical database. As iron-making is a typical process industry, the production data of BF contain rich information and are of strong relevance; thus, the historical production data of BF contain abundant effective information which can be used to guide the operation of BF. However, because of the large amount of data and the lack of an effective data analysis method, the value of historical data has always been ignored by BF operators.

Data mining has been regarded as one of the most promising data analysis approaches in recent years. It emerged as a method for identifying patterns and trends from big data [3, 4]. It includes many algorithms, such as clustering, classification, association rule mining, and regression. Among the many algorithms in data mining, association rule well handles the quantitative data; in addition, the results of association rule mining (ARM) are linguistic and, hence, can be easily understood and could be explained. Nowadays, some ARM algorithms have been widely used in process industry manufacturing, medical science, machine fault detection, and economics to discover useful knowledge, for example, Apriori [5], Fp-tree [6], and Eclat [7].

Regarding the application of ARM in BF, Fei and Chang-Xiu and Guo et al. applied the conventional ARM to BF to discover interesting knowledge for facilitating the stable operation of BF [8, 9]. Although the above methods can find out relevant knowledge of stabilizing furnace conditions, the knowledge obtained by the above methods has limitations in application because of the characteristics of multivariable coupling and large time delay of BF. The TAR algorithm can find the temporal relations among the multivariables well, and the rules can play a better role in stabilizing the furnace state. Therefore, this article proposed a TAR algorithm based on UDP. Compared with TAR algorithms which have been applied in BF before, the algorithm proposed can find the implicit knowledge that other algorithms cannot find. The rules obtained by the proposed algorithm include temporal relation among multivariables, and the number of effective rules obtained is more than that of other algorithms. In addition, the updating algorithm proposed can update the rules in dynamic database fast, which only scan the new transactions and find the frequent itemsets and the itemsets satisfying the UDP condition.

The remainder of this article is organized as follows: some related works are given in Section 2. We introduce background knowledge of this article in Section 3. In Section 4, we discuss the one-dimensional TARs and multidimensional TARs in detail and introduce the UDP method briefly. The proposed algorithm and TAR updating method are also presented in Section 4. A simple example is used to demonstrate the mining process of the proposed algorithm. In Section 5, we use authentic BF data to evaluate the performance of the proposed algorithm. The conclusions of this study are presented in Section 6.

## 2. Related Work

TARs can discover the knowledge that contains temporal information among multiple variables, and studies on TAR mining have been conducted. In [10], Lin et al. extracted frequent patterns from calendar schemes. Then, an *apriori* − *based* algorithm was applied to generate the time association rules. In [11], Chen et al. applied the membership function in fuzzy theory to association rule mining. The method reflects the life span of an item by redefining the *Support* and *Confidence*. The algorithm obtains the effective time of rules through the life span of each item. In addition, Chen et al. proposed a fuzzy time-series mining algorithm. The algorithm can mine the TARs with a sliding window effectively; however, the mining results depend on the window size, and the type of membership function is difficult to specify [12]. Gan et al. proposed two tree-based algorithms for mining the frequent temporal patterns, which consider not only the Support of patterns but also their weights [13]. In [14], Ghorbani and Abessi proposed the concept of a time cube and applied the Apriori algorithm to mine the temporal association rules. However, these methods did not consider multiple items between intertransactions, and the inherent information was difficult to mine, which caused the rules to be less interpretable. Although these rules contain temporal information, they cannot overcome the multiple variable coupling in BF.

For mining intertransactional association rules, a compact FP tree-based divide-and-conquer algorithm was presented by Qin and Shi. The rules that were generated by this algorithm were interpretable; however, the algorithm was sensitive to the parameter values [15]. Ruan et al. presented a framework, which enables parallel and quantitative mining of sequential patterns [16]. Hong et al. proposed the concept of up-to-date patterns, which can mine implicit patterns effectively [17]. Both of these methods can mine only frequent temporal patterns and not association rules. Wang et al. proposed the frequent itemset tree. The algorithm can discover the temporal association rules among multiple items; however, the form of the rules was effective only within a period of time [18, 19]. In [20], Mao considered the problem of efficiently mining association rules in large sample databases and mined TARs from a traffic data set. However, all the rules that were mined in this study are association rules that are based on time constraints, which can be used to discover the life spans of association rules. However, when we apply the association rules to BF, the association rules with valid life spans cannot play a strong role in facilitating decision-making. Since, the association rules with valid time spans cannot provide an exact time point, but a time range, the reference is not useful if the time range is too large for decision makers. Such rules are suitable for regular occurrences of events but are not suitable for BF. Thus, the rules can describe the relevant changes of the system state after time *T* as multiple variables operate simultaneously.

In [21], Tan et al. proposed a mining framework for mining TARs from stock time-series data. However, the method can be sensitive to the size of the sliding window. In [22], Sornalakshmi et al. proposed an algorithm, namely, TAR-IMF, to mine TARs from time-series data. The TAR-IMF algorithm can reduce the execution time and the memory usage. In [23], Dang et al. proposed a novel lattice structure for extracting rules from cancer data and yield satisfactory results. However, the algorithm can only discover the temporal pattern and not association rules. In [24], Khen and Simon proposed a novel method for discovering the temporal association rules; however, the performance of the algorithm is sensitive to a parameter, namely, the temporal-association accuracy (TAA). In [25], Wen et al. used an Apriori-based method to mine TARs and used rules to predict the traffic congestion and obtain a satisfactory result. In [26], Martínez-Ballesteros et al. proposed a quantitative TAR algorithm named QARGA. The QARGA algorithm has two obvious advantages. First, it does not perform a previous attribute discretization, and, second, it does not need to set which variables are antecedent or consequent. The algorithm performs well in discovering TARs in time-series data. In [27], Martínez-Ballesteros et al proposed quantitative association rules based on evolutionary computation techniques. This article proposed to use a real-coded genetic algorithm to determine the intervals that define the rules without needing to discretize the attributes. The proposed method solves the problem of attribute partition in TARs and improves the quality of rules. In [28], Moslehi et al. proposed a new hybrid framework called GA-PSO framework to determine the threshold value in TAR mining. This novel framework is of great significance to solve the subjective problem of threshold determination in TARs and to improve the quality of the obtained rules.

All the TAR algorithms that are discussed above can discover knowledge with generality; however, these studies did not consider that patterns may only appear in a limited time period. These approaches cannot discover these frequent patterns; hence, some implicit knowledge cannot be discovered in the form of TARs. Moreover, this implicit knowledge may more comprehensively reflect the temporal relationships of multiple variables in BF and may play important roles in facilitating decision-making and in stabilizing furnace conditions. To overcome the shortcomings of the TAR algorithm mentioned before, this article proposes an algorithm which can discover the implicit knowledge based on the previous works. Moreover, the rules obtained by the proposed algorithm contain the temporal relations among multivariables.

In this article, a new TAR mining framework is proposed for mining TARs with generality. Moreover, a novel algorithm based on UDP is proposed for discovering the implicit knowledge that cannot be discovered by previous approaches. Applying the proposed framework and TAR mining algorithm to BF data, temporal relationships among multiple variables can be identified in the form of TARs. *Lift* and *CF* are applied to ensure the validity of the rules that are mined by our algorithm. Considering the dynamic updating of BF data, knowledge that we learn from rules may not be applicable to the current furnace conditions. The out-of-date knowledge may lead to drastic changes in furnace conditions and cause abnormal furnace conditions. Therefore, we also propose methods for identifying outdated rules and dynamically updating rules.

Briefly, we make the following contributions:

- We propose using TARs to discover knowledge that will contribute to the stability of the conditions of BF. Our proposed method not only discovers the temporal relations among multiple variables in BF but also uncovers the implicit knowledge.
- We propose a new TAR mining framework and a novel algorithm for mining implicit knowledge. Moreover, to discover the up-to-date knowledge, the concept of *Recency* is used to identify out-of-date TARs.
- We develop a TAR updating method for maintaining TARs in a dynamically updated BF database. And, the proposed updating method can mine implicit knowledge while updating TARs.

## 3. Preliminaries

In this section, we define variables and concepts that are used in our study.

### 3.1. Association Rule Mining

**Definition 1 .**

*Support*(*X*) describes the probability of transaction *X* appearing in *D*:

(1) $$\begin{array}{c} Support\left(X⟶Y\right)=\frac{Count\left(X\right)}{\left|D\right|}, \end{array}$$

where *Count*(*X*) is defined as the total number of times that transaction *X* appears in the log database and |*D*| is the total number of transactions in the log database.

**Definition 2 .**

*Support*(X ⟶ Y) describes the probability of transactions *X* and *Y* both appearing in *D*:

(2) $$\begin{array}{c} Support\left(X⟶Y\right)=\frac{F\left(X,Y\right)}{\left|D\right|}, \end{array}$$

where *F*(*X*, *Y*) is defined as the total number of times that transactions *X* and *Y* appear at the same time.

**Definition 3 .**

*Confidence*(*X* ⟶ *Y*) describes the probability of *X* appearing under the condition of *Y*:

(3) $$\begin{array}{c} Confidence\left(X⟶Y\right)=\frac{Support\left(X⟶Y\right)}{Support\left(X\right)}. \end{array}$$

In ARM, min_sup and min_conf are two important parameters: min_sup denotes the frequency of itemsets and min_conf denotes the reliability of rules. Because rules with low *Confidence* are not credible, min_conf is typically greater than 0.6 and less than 1.

**Definition 4 .**

Downward property: If *Y* is frequent (*Support*(*Y*) ≤ min_sup), then any subset *X* of *Y* is also frequent because (*Support*(*X*) ≤ *Support*(*Y*) ≤ min_sup) [23].

### 3.2. Rule Evaluation

Several authors have identified drawbacks of the *Support* and *Confidence* framework for assessing association rules [29].

To avoid some of these drawbacks and to ensure that the rules that are mined by our algorithm are accurate and relevant, researchers proposed the concept of *Lift* for ensuring that the rules are useful. A rule is meaningful only if its *Lift* value is greater than 1 and a meaningful rule is called a strong association rule. The formula for *Lift* is as follows:

(4) $$\begin{array}{c} Lift\left(X⟶Y\right)=\frac{Support\left(X\cup Y\right)}{Support\left(X\right)*Support\left(Y\right)}=\frac{P\left(\left.Y\right|X\right)}{P\left(Y\right)}. \end{array}$$

Moreover, a new approach for evaluating rules was proposed in [30, 31]. A new concept, namely, the certain factor (*CF*), is employed.

**Definition 5 .**

*CF*(*X*⟶*Y*) is the certain factor of association rules *X*⟶*Y* to the following value:

(5) $$\begin{array}{c} CF\left(X⟶Y\right)=\frac{Confidence\left(X⟶Y\right)-Support\left(Y\right)}{1-Support\left(Y\right)}, \end{array}$$

if *Confidence*(*X*⟶*Y*) > *Support*(*Y*) and

(6) $$\begin{array}{c} CF\left(X⟶Y\right)=\frac{Confidence\left(X⟶Y\right)-Support\left(Y\right)}{Support\left(Y\right)}, \end{array}$$

if *Confidence*(*X*⟶*Y*) ≤ *Support*(*Y*).

Assume by agreement that if *Support*(*Y*)=1, then *CF*(*X*⟶*Y*)=1, and if *Support*(*Y*)=0, then *CF*(*X*⟶*Y*) is −1.

The *CF* yields a value in the interval [−1, 1] and measures how we believe *Y* in a transaction changes when we are told that *X* is in the transaction. If *CF* > 0, then the rule has been confirmed by observed evidence; if *CF* < 0, then the evidence lends credence to the negation of the rule; and if *CF*=0, then there is no evidence that supports the rule.

In this article, to ensure the accuracy and relevance of the rules, we shall use *Lift* and *CF* to measure the accuracy of the rules. A rule is referred to strong if its *CF*, *Lift*, and *Confidence* all exceed the user-defined thresholds.

### 3.3. Recency of Rules

Association rules that are discovered in a temporal database may change over time. Extracting up-to-date knowledge, especially from a temporal database, can provide valuable and timely information for decision-makers [32]. However, most algorithms that are used to mine TARs from temporal databases do not consider the recency of the rules. In this section, we will define Recency briefly and derive in detail the time decay function that is used in this article.

**Definition 6 .**

The *Recency* of itemset *X* in *T* is denoted as *R*(*X*, *T*) and defined as [32]:

(7) $$\begin{array}{c} R\left(X,T\right)=R\left(T\right). \end{array}$$

**Definition 7 .**

The *Recency* of itemset *X* in *D* is denoted as *R*(*X*) and defined as [32]:

(8) $$\begin{array}{c} R\left(X\right)=\sum R\left(X,T\right), \end{array}$$

where *X*⊆*T*Λ*T* ∈ *D*. *R*(*X*, *T*) and *R*(*X*) can be calculated via a time decay function.

To identify out-of-date rules that have been mined by the TAR mining algorithm, we introduce a time decay function for assigning a recency weight to each transaction. A transaction is assigned a higher recency weight if it is temporally close to the current transaction. In this article, we use Newton's law of cooling to establish the time decay function (other reasonable time functions can also be used.).

Newton's law of cooling can be briefly summarized as follows: the cooling rate of a body is proportional to the difference between the current temperature and the room temperature. This can be expressed mathematically as follows:

(9) $$\begin{array}{c} T{\left(t\right)}^{\prime }=-\alpha \left(T\left(t\right)-H\right), \end{array}$$

where *α* is the attenuation coefficient and *H* is the current temperature.

Next, we will use formula (9) to derive the time decay function. Formula (9) can be reexpressed as

(10) $$\begin{array}{c} \frac{T{\left(t\right)}^{\prime }}{T\left(t\right)-H}=-\alpha . \end{array}$$

Integrating both sides of formula (10) yields

(11) $$\begin{array}{c} \int \frac{T{\left(t\right)}^{\prime }}{T\left(t\right)-H}\text{d}t=\int \alpha \text{ d}t. \end{array}$$

Solving the differential equation in formula (11) yields

(12) $$\begin{array}{c} \ln\left(T\left(t\right)-H\right)=-\alpha t+c. \end{array}$$

Thus, we can derive the function *T*(*t*):

(13) $$\begin{array}{c} T\left(t\right)=H+c\cdot \;\exp\left(-\alpha t\right). \end{array}$$

In this article, we will use formula (13) as the time decay function. Now, we must determine the values of *H* and *c* in formula (13). Let *t*=|*D*| − *Trans*_*i*, where |*D*| denotes the total number of transactions in the database and *Trans*_*i* is the order of the transactions in the database. The smaller the |*D*| − *T* is, the closer the transaction is to the current transaction, namely, the greater the *Recency*. If *t*=0, then *T*=1; thus, *c*=1 − *H*. As *t* approaches positive infinity, *T*(*t*) should converge to 0, hence, *H*=0 and *c*=1. Finally, the time decay function that we use in this paper can be expressed as

(14) $$\begin{array}{c} T\left(t\right)=\exp\left(-\alpha t\right), \end{array}$$

where *α* is the attenuation coefficient and satisfies *α* > 0, which is determined by users.

To identify the out-of-date rules with the time decay function, a minimum threshold for *min*_*Recency* could be set. If we identify *N* transactions and the latest 30% of these transactions are considered recent, the sum of the function values of these transactions can be calculated as

(15) $$\begin{array}{c} S_{n}=1+\exp^{a}+\exp^{2*a}+\cdots +\exp^{a*0.3N}\\ =\exp^{0*a}+\exp^{1*a}+\exp^{2*a}+\cdots +\exp^{a*0.3N}. \end{array}$$

Formula (16) can be obtained from the formula of equal ratio:

(16) $$\begin{array}{c} S_{n}=\frac{1-\;\exp^{-\alpha *0.3N}}{1-\;\exp^{-\alpha }}. \end{array}$$

We conclude that *S*_*n*_ is a constant that is associated with the attenuation coefficient *α* and the number of transactions *N*. Because of the characteristics of the time delay function that is used in this article, the function value is less than or equal to 1; moreover, the function is a monotone decreasing function, namely, if a rule is out-of-date (the rule does not appear in the latest 30% of transactions but only appear in the other 70% of transactions), then the function value of this rule will be very small, and may be close to 0. By contrast, if a rule is up-to-date and satisfies rule mining conditions, its function value is relatively large. The *Recency* of a rule must exceed min_Recency for the rule to be considered as an up-to-date rule.

## 4. The Proposed Method for Mining Association Rules for BF Application

In this section, a new TAR mining framework and a novel TAR algorithm are proposed for discovering underlying knowledge. Because the database of BF is dynamically changing, a TAR updating method is also proposed for maintaining TARs.

### 4.1. Temporal Association Rule Mining

For mining interesting patterns from time series, a conventional association rule mining algorithm (such as: Apriori, FP-Growth, and Eclat) can only identify some frequent patterns without time constraints. As we discussed, these rules are often duplicated with expert knowledge; hence, their reference value is not high. Moreover, they cannot express the relations of variables on a time scale. Thus, it is crucial to discover a new association rule mining framework that considers time information based on the relationships among multiple variables. To overcome this drawback of the classical *Support* and *Confidence* framework, we propose a new TAR mining framework for mining TARs in the form of $X\overset{T}{⟶}Y$. Next, we will divide TARs into one-dimensional TARs and multidimensional TARs to clarify the new framework of TARs.

#### 4.1.1. One-Dimensional TARs

One-dimensional TARs can be briefly described as follows: if *X* occurs at time *t*, then *Y* will appear at time *t* *+* *T*. The form of rule can be expressed as: $\text{Rule}\left(X\overset{T}{⟶}Y\right)$.

**Definition 8 .**

*Support* $\left(X\overset{T}{⟶}Y\right)$ describes the probability of both transaction *X* appearing in *D* at time *t* and transaction *Y* appearing in *D* at time *t*+*T*:

(17) $$\begin{array}{c} Support\left(X\overset{T}{⟶}Y\right)=\frac{F\left(X,Y,T\right)}{\left|D\right|-T}, \end{array}$$

where *F*(*X*, *Y*, *T*) is defined as the total number of transactions that satisfy the following: if *X* appears at time *t*, then *Y* appears at time *t* *+* *T*. |*D*| is the total number of transactions in the log database.

And, from the classical framework, we can get the definition of Confidence in one-dimensional TARs:

**Definition 9 .**

*Confidence* $\left(X\overset{T}{⟶}Y\right)$ describes the probability of transaction *X* appearing in *D* at time *t* under the condition that *Y* appears in *D* at time *t*+*T*:

(18) $$\begin{array}{c} Confidence\left(X\overset{T}{⟶}Y\right)=\frac{Support\left(X\overset{T}{⟶}Y\right)}{Support\left(X\right)}. \end{array}$$

The sequence of the items is not considered in traditional methods; however, the sequence of the items must be considered in TARs. Therefore, the method for generating candidate itemsets in TARs has been changed, and we will present the algorithm for generating candidate itemsets in one-dimensional TARs as Algorithm 1.

#### 4.1.2. Multidimensional TARs

Aiming at discovering relationships among multiple items with time constraints, multidimensional TARs are proposed. Briefly, multidimensional TARs can be described as follows: if *X*_1_, *X*_2_,…, *X*_*m*_ appear at time *t* and *Y*_1_, *Y*_2_,…, *Y*_*n*_ appear at time *t*+*T*, then the rule can be expressed as

(19) $$\begin{array}{c} Rule:X_{1}\wedge X_{2}\wedge X_{3}\wedge ,\ldots ,\wedge X_{m}\\ \overset{T}{⟶}Y_{1}\wedge Y_{2}\wedge Y_{3}\wedge ,\ldots ,\wedge Y_{n}. \end{array}$$

**Definition 10 .**

*Support*(*X*_1_, *X*_2_,…, *X*_*m*_) describes the probability of transactions *X*_1_, *X*_2_,…, *X*_*m*_ appearing in *D* simultaneously:

(20) $$\begin{array}{c} Support\left(X_{1}\wedge X_{2}\wedge ,\ldots ,\wedge X_{m}\right)=\frac{F\left(X_{1}\wedge X_{2}\wedge ,\ldots ,\wedge X_{m}\right)}{\left|D\right|}, \end{array}$$

where |*D*| denotes the total number of transactions in log database *D*.

**Definition 11 .**

$Support\left(X_{1},X_{2},\ldots ,X_{m}\overset{T}{⟶}Y_{1},Y_{2},\ldots ,Y_{n}\right)$ describes the probability of transactions *X*_1_, *X*_2_,…, *X*_*m*_ appearing in *D* at time *t* and transactions *Y*_1_, *Y*_2_,…, *Y*_*n*_ appearing in *D* at time *t*+*T*:

(21) $$\begin{array}{c} Support\left(X_{1}\wedge X_{2}\wedge ,\ldots ,\wedge X_{m}\overset{T}{⟶}Y_{1}\wedge Y_{2}\wedge ,\ldots ,\wedge Y_{n}\right)\\ =\frac{F\left(X_{1}\wedge X_{2}\wedge ,\ldots ,\wedge X_{m},Y_{1}\wedge Y_{2}\wedge ,\ldots ,\wedge Y_{n},T\right)}{\left|D\right|-T}, \end{array}$$

*F*(*X*_1_∧*X*_2_∧,…, ∧*X*_*m*_,  *Y*_1_∧, *Y*_2_∧,…, ∧*Y*_*n*_, *T*) in formula (21) is the total number of transactions that satisfy the requirement that if *X*_1_∧*X*_2_∧,…, ∧*X*_*m*_ appear at time *t*, then *Y*_1_, *Y*_2_,…, *Y*_*n*_ appear at time *t*+*T* simultaneously.

**Definition 12 .**

$Confidence\left(X_{1},\ldots ,X_{m}\overset{T}{⟶}Y_{1},\ldots ,Y_{n}\right)$ describes the probability of transactions *Y*_1_,…, *Y*_*n*_ appearing in *D* at time *t*+*T* under the condition that *X*_1_,…, *X*_*m*_ appear in *D* at time *t*:

(22) $$\begin{array}{c} Confidence\left(X_{1}\wedge X_{2}\wedge ,\ldots ,\wedge X_{m}\overset{T}{⟶}Y_{1}\wedge Y_{2}\wedge ,\ldots ,\wedge Y_{n}\right)\\ =\frac{Support\left(X_{1}\wedge X_{2}\wedge ,\ldots ,\wedge X_{m}\overset{T}{⟶}Y_{1}\wedge Y_{2}\wedge ,\ldots ,\wedge Y_{n}\right)}{Support\left(X_{1}\wedge X_{2}\wedge ,\ldots ,\wedge X_{m}\right)}. \end{array}$$

Formula (22) can be calculated by using formulas (20) and (21).

As we discussed for one-dimensional TARs, the order of the items in TARs must be considered, in multidimensional TARs. Thus, the method of generating candidate *k*-itemsets *C*_*k*_ differs from the traditional method because the downward property cannot be ensured by the traditional method. To ensure the downward property, we proposed an algorithm for generating candidate itemsets in Algorithm 2.

To describe Algorithm 2, we rewrite the frequent itemsets *L*_*k*_ and candidate itemsets *C*_*k*_ in a more specific way. For example, if we have a frequent 2-itemsets (*a*, *c*), as discussed earlier, the sequence of items must be considered. Item *a* is the antecedent and item *c* is the consequent; hence, we rewrite the frequent 2-itemsets (*a*, *c*) as (*a*⟶*c*). According to the representation of frequent items *L*_*k*_, we classify each itemsets in *L*_*k*_ into two parts: the part on the left side of the arrow is classified as Class_1_, and the part on the right side of the arrow as Class_2_. As a result, the candidate *k* itemsets can be obtained via Algorithm 2.

#### 4.1.3. Rule Generation

In the traditional association rule mining algorithm, after identifying frequent patterns, a rule is generated if it satisfies the *Confidence* threshold without considering the order of the items. Because the time constraints are considered, the process of rule generation differs from the traditional processes. Moreover, to determine the out-of-date rules, the min_Recency has been integrated into the rules generation process. The generation of TARs can be realized via Algorithm 3.

### 4.2. Up-To-Date Patterns

Usually, the mining association rules from the log database can be summarized into two parts:

1. Find all frequent items in the original log database according to the predefined min_sup
2. Generate association rules in frequent items according to the predefined min_conf

Frequent itemsets are certified by min_sup, namely, if an itemset appears frequently in the log database, it is considered frequent. However, some itemsets only appear frequently in a limited period of time but not for the whole database; the traditional min_sup threshold is inadequate for mining such frequent itemsets. However, these implicit frequent itemsets may play a more important role. To address this problem, we combine the Apriori algorithm with up-to-date patterns (UDPs) to discover this underlying knowledge in the form of TARs.

Hong et al. proposed the concept of UDP, which were frequent within their up-to-date lifetimes. Lin et al. proposed an algorithm for deriving up-to-date patterns from transactions [33, 34]. An advantage of the UDP method is that it can mine the implicit frequent patterns that satisfy the current min_sup threshold without changing it. If the min_sup is reduced to mine implicit rules, rules explosion will occur. This method records the occurrence time of each item as *Timelist*(*i*) when scanning the log database and mines the itemsets that do not satisfy the min_sup threshold to discover implicit itemsets via the following formula:

(23) $$\begin{array}{c} n-First\_ID+1\leq \frac{count\left(i\right)}{\mathrm{min\_sup}}, \end{array}$$

where *n* is the total number of transactions in the log database, *First*_*ID* is the first transaction ID in *Timelist*(*i*), *count*(*i*) is the number of occurrences of item *i* in the log database, and min_sup is the minimum *Support*, which is set in advance.

Using formula (23) and the concept of *First*_*ID*, one can reduce the size of the database and determine whether each itemsets is frequent in the reduced database. Via this process, the UDP method can mine the implicit frequent patterns that only appear in a limited period of time.

### 4.3. The Proposed Algorithm

#### 4.3.1. Description

**Figure 1:**
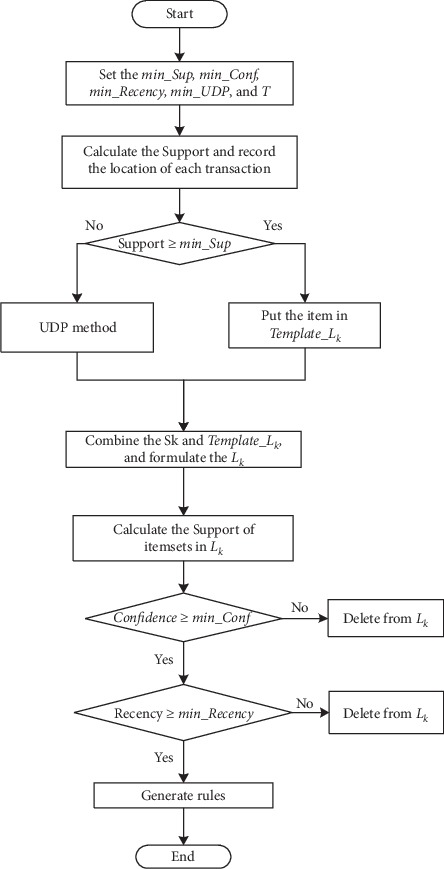
The flowchart of the proposed algorithm.

**Figure 2:**
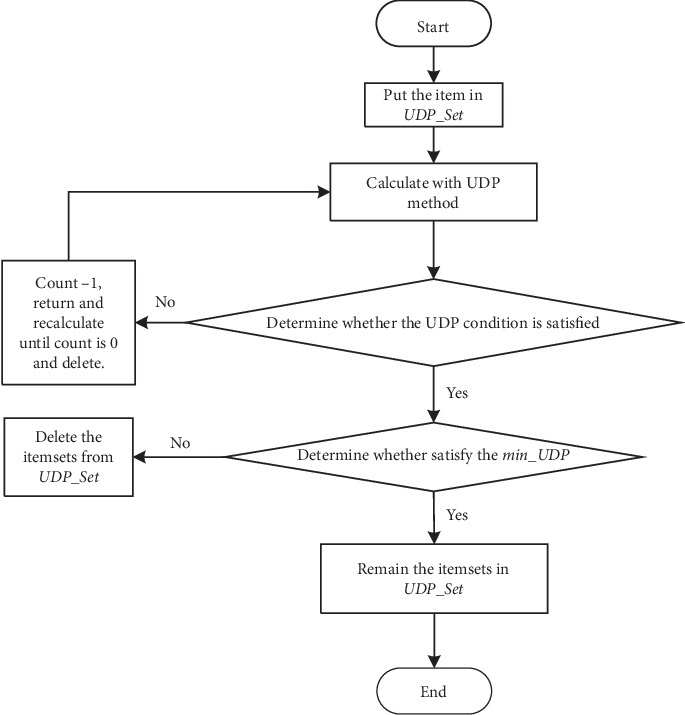
The flowchart of UDP method in Figure 1.

The main objective of the algorithm we proposed in this article is to determine the relationships among multiple variables with time constraints. To discover the implicit information, we combine the Apriori algorithm and the concept of up-to-date patterns. As we discussed above, the original *Support* and *Confidence* of Apriori cannot satisfy the requirements of mining association rules with time constraints from time series; thus, the new *Support* and *Confidence* framework is adapted. The steps of the proposed algorithm will be described in next section and flowcharts are shown in Figures 1 and 2.

**Definition 13 .**

*UDP* _*Set*_*k*_ denotes the *k*-itemsets that cannot be mined by the *Support* framework but can be mined via the UDP method, where *k* denotes that each itemsets contains *k* items.

**Definition 14 .**

The *min*_*UDP* is the parameter for pruning the itemsets that are mined via the UDP method (Figure 2). Its value is greater than 0 and less than min_sup.

As min_UDP increases gradually, the number of itemsets that can be mined via the UDP method decreases.

#### 4.3.2. Construction of Algorithm

Temporal association rule mining with up-to-date patterns.

**Input**: A log database *D* with *n* transactions, each of which includes the transaction ID, the transaction time, and the items. The time *T*, the minimum *Support* threshold min_sup, the minimum *Confidence* threshold min_conf, the minimum *Recency* threshold min_Recency, and the minimum UDP threshold *min_UDP.*
**Output**: The temporal association rules that have been mined from the time series.
**Step 1**: Scan the database *D* to form the candidate 1 − *itemsetC*_1_ and record the *count* value and *Timelist*(*i*) of item *i* in the log database.
**Step 2**: Complete the following substeps for the items in *C*_1_:
   
**Substep 2.1**: Calculate the *Support* of the *i* − th item in *C*_1_.
   
**Substep 2.2**: If the *Support* of the item is more than min_sup, then put the item in *Template* − *L*_1_; otherwise, put the item in *UDP* _*Set*_1_ and jump to Step 3.
**Step 3**: Complete the following substeps for the items in *UDP* _*Set*_1_:
   
**Substep 3.1**: For the items in *UDP* _*Set*_1_, set the *First*_*ID*(*i*) as the first transaction ID in *Timelist*(*i*) of item *i* and determine whether item *i* satisfies formula (23). If item *i* satisfies formula (23), then jump to STEP 4; otherwise, jump to Substep 3.2.
   
**Substep 3.2**: Set *First*_*ID*(*i*) as the next transaction ID in *Timelist*(*i*) of item *i*, decrease the *count* of item *i* by one, and repeat Substep 3.2 until *count*(*i*) is equal to zero. If *count*(*i*) is equal to zero and the item or itemset still does not satisfy formula (23), then it will be deleted from *UDP* _*Set*_1_.
**Step 4**: Determine whether the *Support* of the itemsets exceeds min_UDP. If the Support of the itemsets exceeds min_UDP, it will be retained; otherwise, it will be deleted.
**Step 5**: Combine the set *UDP* _*Set*_1_ and the set *Template* − *L*_1_ to form *L*_1_. Set *r* = 1, where *r* is used to keep the current number of items in the itemsets to be processed.
**Step 6**: Generate candidate set *C*_*r*+1_ from *L*_*r*_ via the method of Algorithm 1 or Algorithm 2. Algorithm 1 can be used when *r*=1.
**Step 7**: Generate the frequent (*r* *+* 1)-patterns (*L*_*r*+1_) from *C*_*r*+1_ via a similar approach to that in STEP 2 to STEP 4.
**Step 8**: If *L*_*r*+1_ is null, continue to the next step; otherwise, jump to STEP 6 and STEP 7.
**Step 9**: Calculate the *Confidence* and *Recency* of the itemsets in *L*_*r*_(*r* ≥ 2). If the *Confidence* and the *Recency* of the itemsets exceed min_*conf* and min_Recency, respectively, rules will be generated via Algorithm 3. Otherwise, delete the itemsets that do not satisfy the requirements of min_conf and min_Recency in *L*_*r*_.
**Step 10**: Output the association rules that have been mined from the log database.

Transactions in the log database must be time series with equal intervals.

### 4.4. Examples

To demonstrate the proposed algorithm, an example is presented below.  Table 1 presents the log database, which contains 10 transactions and 6 items with time stamps.

 
**Input**: *T* = 3, min_sup=0.5, min_conf=0.4, min_*UDP*=0.2, log database *D*
 
**Output**: Association rules that have been mined from *D*
 
**Step 1**: Scan the database and find *count(i)* and *Timelist(i*) of item *i* in *D.* Consider item *a* as an example. It appears in seven transactions; hence, *count*(*a*) is 7 and *Timelist*(*a*) is {1, 2, 3, 4, 5, 6, 10}. The results of STEP 1 are shown in Table 2.

 
**Step 2**: Calculate the *Support* in Table 2 via formula (1). Consider item *b* as an example, the *count* of *c* is 5; therefore, according to formula (1), the *Support* of *c* is 0.5. The min_sup value that is specified above is 0.5; hence, *c* will be placed in *Template*_*L*_1_. The *Support* of item *b* is 0.2, which is less than min_sup; therefore, it will be placed in *UDP* _*Set*_1_. The results of the *Support* calculation are listed in Table 3. *Template*_*L*_1_={*a*, *c*, *e*} and *UDP* _*Set*_1_={*b*, *d*, *f*}.

 
**Step 3**: For the items in *UDP* _*Set*_1_, perform the following steps: Consider item *b* and item *d* as examples.
  For item *b*: *Timelist*(*b*)={8,9}; hence, *First*_*ID*(*b*)=8. In addition, *n*=10, *count*(*b*)=2, and min_sup=0.5. Substitute these parameter values into formula (18): on the left side of inequality is 10 − 8+1=3; on the right side is (2/0.5)=4. Therefore, item *b* satisfies formula (23). Proceed to STEP 4.

  For item *d*: the *Timelist*(*d*)={1,2,3}; hence, *First*_*ID*(*d*)=1. Substitute the parameters into formula (23): on the left side of inequality is 10 − 1+1=10; on the right side is (3/0.5)=6. Formula (23) is not satisfied. Therefore, jump to Substep 3.2. The *count*(*d*)=3 − 1=2, and set *First*_*ID*(*d*)=2. Substitute the updated parameters into the inequality and recalculate. The result still does not satisfy the inequality. Repeat Substep 3.2, the *count*(*d*)=2 − 1=1, and the *First*_*ID*(*d*)=3; substitute the updated parameters to the inequality and recalculate, the result still cannot satisfy the inequality. Repeat Substep 3.2. Because *count*(*d*)=0, delete item *d* from *UDP* _*Set*_1_.

 
**Step 4**: If the item satisfies formula (23), then determine whether the Support of the item is greater than min_UDP. The Support of item *b* is 0.2, which satisfies the min_UDP requirement; thus, it will remain in *UDP* _*Set*_1_.
 
**Step 5**: Combine *UDP* _*Set*_1_ set *Template*_*L*_1_ to form *L*_1_={*a*, *b*, *c*, *e*, *f*}, and set *r*=1.
 
**Step 6**: Generate the candidate set *C*_2_ from *L*_1_ via Algorithm 1. *C*_2_ is presented in Table 4.

 
**Step 7**: Generate the frequent 2-patterns *L*_2_ via a similar approach to that in STEP 2 to STEP 4. The *Template*_*L*_2_ is (*a*⟶*e*) and *UDP* _*Set*_2_ is null. Therefore, *L*_2_=(*a*⟶*e*).
 
**Step 8**: Because there is only one itemset in *L*_2_, the candidate 3-itemsets *C*_3_ is null and the algorithm proceeds to STEP 9.
 
**Step 9**: Calculate the *Confidence* of $a\overset{T}{⟶}e$ via formula (13). $Support\left(a\overset{T}{⟶}e\right)$ is 0.5 and *Support*(*X*) is (7/10). Therefore, $Confidence\left(a\overset{T}{⟶}e\right)$ is (5/7), which satisfies the min_conf threshold, and the algorithm run to STEP 10.
 
**Step 10**: The rule can be generated via Algorithm 3. *Class*_1_ of *L*_1_ is item *a* and *Class*_2_ of *L*_1_ is item *e.* Therefore, we can get the rule: $Rule=a\overset{T}{⟶}e$ with *Confidence*=(5/7).

### 4.5. Rule Updating

Because the BF database is updated dynamically, facing the dynamic database, most algorithms must rescan the whole database to generate new association rules. Such methods do not consider the values of frequent item sets that have been mined previously and are time consuming. To maintain TARs that were mined from the BF database, we propose a method for rapidly updating TARs in dynamically updated temporal databases.

**Figure 3:**
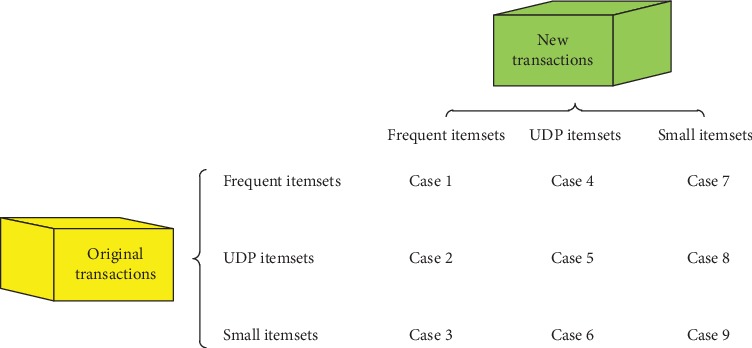
Nine cases of database update.

In the past, to mine association rules in dynamic databases, Cheung et al. proposed the FUP algorithm for effectively handling new transactions for maintaining association rules [32]. Moreover, aiming at updating association rules in a dynamic database, Hong and Lin et al. propose the FUFP and FUFP-tree algorithms for solving transaction insertion and deletion from the database [35–38]. To maintain TARs that are mined by the proposed algorithm in a dynamic database, we combine Pre-FUFP with the proposed algorithm in this article. The main strategy of the Pre-FUFP algorithm [39] is as follows: considering original transactions and transactions which are newly inserted, an itemset may fall into one of the nine cases in Figure 3.

In Figure 3, there are three types of itemsets: frequent itemsets, UDP itemsets, and small itemsets. Frequent itemsets are the itemsets that satisfy the min_sup threshold. UDP itemsets are itemsets that do not satisfy the min_sup threshold but can be mined via the method that is proposed in this article. Small itemsets are itemsets that neither satisfy the min_sup threshold nor can be mined via the algorithm that is proposed in this article.

In Table 5, the results of various cases are listed. Case 1 and case 9 will not affect the final association rules. In cases 2, 3, 4, and 5, it is necessary to rescan the updated database to determine whether the itemsets are frequent; however, due to the property of up-to-date patterns that was discussed in the previous section, the itemsets that correspond to these four cases must be UDP-Sets. In case 3, although each itemset is small in the original database, it may be a UDP-Set because of its frequency in the new transaction database. Because cases 3, 6, 7, and 8 may remove or add new association rules, the updated database must be rescanned. The itemsets that correspond to case 6 may not satisfy the min_UDP threshold and will no longer be UDP-Sets; thus, the itemsets that correspond to case 6 must be rescanned. With the Pre-FUFP algorithm, when new transactions are obtained, we can identify the new *Frequent*_*itemsets*, *UDP* − *Sets*, and *Small*_*itemsets* by scanning the new transactions.

In this article, to maintain association rules in a dynamic database, we proposed an algorithm for fast rule updating, which is presented in Algorithm 4. When new transactions arrived, we only scan the new transactions and find the frequent itemsets and the itemsets that satisfy the UDP condition, which we refer to as *New*_*Frequent* and *New*_*UDP*, respectively. Drawing *support* from previous mining process information, we can identify the frequent itemsets and UDP itemsets in the original database. We combine frequent itemsets and UDP itemsets in the original database and new transactions, delete duplicate itemsets, and refer to the merged itemsets as *candidate*_*sets*. Now, we only need to determine whether the itemsets in the *candi* *date*_*sets* satisfy the *Support* and UDP requirements in the algorithm that we proposed. To evaluate the performance of the algorithm we proposed, we will compare it with the FUFP algorithm, and the results will be presented in the next section.

## 5. Application in Blast Furnace Iron-Making

In this section, we apply the proposed algorithm to the real BF production data to mine TARs. At the same time, we use *Lift* and *CF* to ensure the validity of the mining rules and the rules, along with the *Recency* value, to ensure that the rules are still applicable to the current situation.

### 5.1. Algorithm Evaluation

BF is a typical process industry, namely, its production data are sequential, which satisfies the mining conditions of the algorithm that is proposed in this article. The proposed method will be applied to mine the TSAR from the authentic blast furnace data of a steel plant in China. The data are discrete time series with a sampling time of 30 min. Based on previous research on BF, we choose 11 variables as input of the algorithm. (Although only 11 variables in BF are selected, the algorithm that is proposed in this article can also run when more variables are selected for other data sets.) The data must be discretized. An intuitive method is to divide the range of quantitative attributes into finite intervals of assigned symbols to form <*attributes, interval*> pairs. According to expert knowledge, they should be divided into three states: descent, normal fluctuation, and ascent. The input variables and the corresponding discretization intervals are presented in Table 6.

The interval division and coding are presented in Table 7 and the variable coding in Table 8. To demonstrate further, we present a simple example. Assume that the blast volume is 3450, as specified in Tables 6 and 7. If the blast volume is less than 3400, then it corresponds to descent and is encoded as 1, 3400 ~ 3500 is encoded as 2, and more than 3500 belongs to ascent and is encoded as 3. In addition, each input is encoded according to the order of the data in Table 8. For example, the blast volume is 1. Therefore, blast volume of 3450 will be encoded as 12, where the former digit represents the blast volume and the latter digit represents normal fluctuation. According to the above method, all blast furnace data can be discretized and used as the input of the algorithm in this article.

To evaluate the performance of the algorithm that is proposed in this article, all experiments were performed in MATLAB 2017a on a personal computer, with a 2.5 GHZ Core CPU. We selected 1438 data from an authentic blast furnace as sample data for time-series association rule mining. To evaluate the performance of the proposed algorithm, we will compare it with the performances of the state-of-the-art algorithms [23, 24] and FP-Growth.

**Figure 4:**
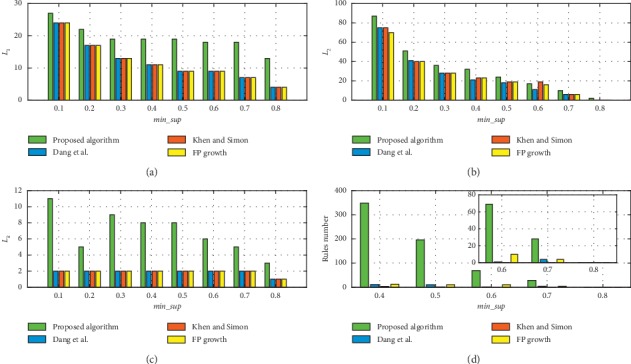
Rules number, *L*_1_, *L*_2_, and *L*_*k*_ comparison when min_conf is 0.6, min_*CF* is 0.4, *T*=6.

In Figure 4, we compare the *L*_1_, *L*_2_, *L*_*k*_, and the number of rules that are mined by the proposed algorithm with the corresponding state-of-the-art algorithms [23, 24] and FP-Growth when min_conf is 0.6, *min*_*CF* is 0.4, and *T* = 6. In this experiment, the values of min_sup ranged from 0.1 to 0.8. When min_sup exceeds 0.9, all algorithms cannot extract rules. In Figure 4, *L*_1_ and *L*_2_ are frequent itemsets. The more the number of *L*_1_ and *L*_2_, the more frequent patterns can be mined and more knowledge can be discovered in the form of TARs. And *L*_*k*_ is the maximum number of items in the rules. A TAR contains more items if the temporal relation among these items can be mined. As we discussed previously, for expert experience, it is difficult to discover the temporal relations among multiple items. With the proposed algorithm, temporal relations among multiple items can be discovered efficiently. Finally, the size of TARs can be expressed as the amount of knowledge available, which can play a role in facilitating decision-making.

The comparison results of *L*_1_, *L*_2_, and *L*_*k*_ are presented in Figures 4(a)–4(c), respectively. The sizes of *L*_1_ that are mined by the proposed algorithm are larger than those of the other methods. The main reason is that some items may appear in limited time period but not over the whole time and, the other methods cannot effectively mine such frequent itemsets; hence, the proposed method can discover more implicit frequent patterns. According to Figure 4(c), the *L*_*k*_ comparison results in Figure 4(c), and the proposed algorithm can identify the temporal relationships among multiple variables which means significant to BF operation. According to the comparison result that is presented in Figure 4(d), more rules were mined by the proposed algorithm than by the other methods, even when min_sup is high, which further demonstrates the superior performance of the proposed algorithm. Thus, the algorithm that we proposed in this article can not only mine relationships among multiple variables but also discover more useful rules. Therefore, the algorithm that we propose will be more suitable for BF operation decision-making.

**Figure 5:**
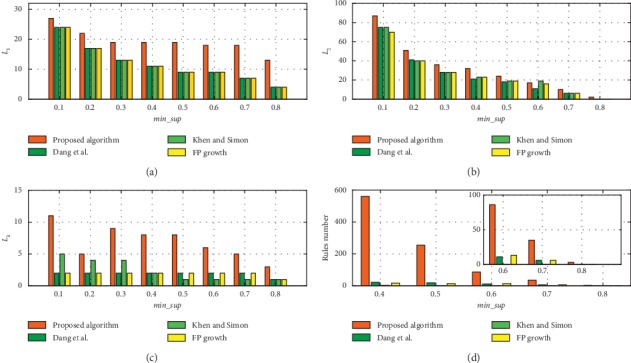
Rules number, *L*_1_, *L*_2_, and *L*_*k*_ comparison without min_CF threshold when min_conf is 0.6, *T*=6.

Because *CF* is applied to further evaluate the correctness of rule mined by algorithm, in Figure 5, we show the comparison results of frequent itemsets and rules number without min_*CF* threshold. Comparing with Figure 5, we can figure out *CF* does filter some noninteresting or nonuseful rules. Applying min_*CF* threshold we can further confirm the validity of the proposed algorithm and the correctness of rules.

**Figure 6:**
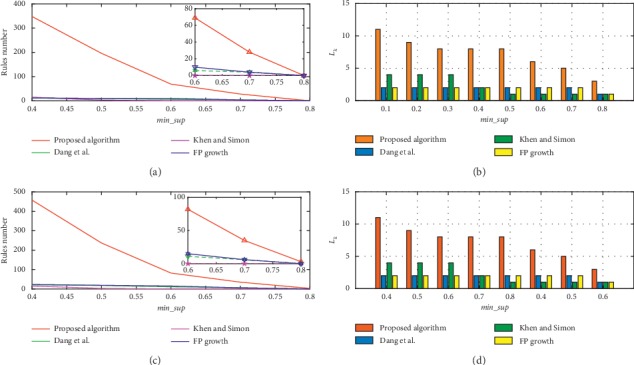
Rules number, and *L*_*k*_ comparison with or without min_CF threshold when min_*conf* is 0.7, *T*=6.

**Figure 7:**
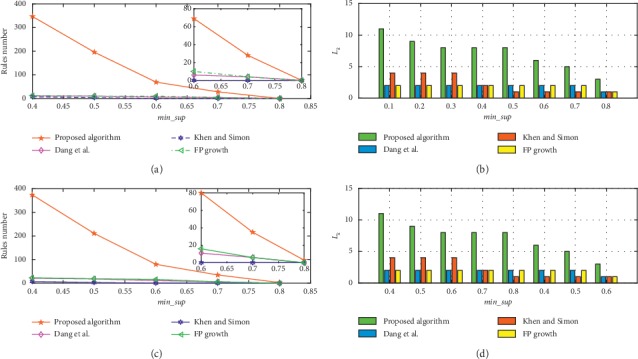
Rules number, and *L*_*k*_ comparison with or without min_CF threshold when min_c*onf* is 0.8, *T*=6.

To evaluate the performance of the proposed algorithm under higher min_*conf*, we compare the mining results of four methods with min_*conf* values of 0.7 and 0.8, as plotted in Figures 6 and 7. Because min_sup varies from 0.1 to 0.8, the mining result comparisons of *L*_1_ and *L*_2_ are consistent with Figures 4(a) and 4(b).

Figures 6(a) and 6(b) show the comparison results for the number of rules and *L*_*k*_ using the min_*CF* threshold, and the results for the number of rules and *L*_*k*_ without using min_*CF* are presented in Figures 6(c) and 6(d). Similar to Figures 6, in 7, we compare the results for the number of rules and *L*_*k*_ when min_conf is set to 0.8, *T*=6. Comparison results with min_*CF* is presented in Figures 7(a) and 7(b). In Figures 7(c) and 7(d) are the results without using *min*_*CF*. According to Figures 6 and 7, the algorithm that is proposed in this article outperforms the other methods when the min_conf is high. Because a rule with low *confidence* is not credible, the rule with lower *Confidence* than 0.6 will not be considered in this article.

Because *T* is a constant value that is selected in advance, in Table 9, we list the number of rules that can be mined via the proposed method with various values of *T*. The proposed algorithm can be well adapted to various *T* values, and the choice of *T* value depends on the temporal information to be mined.

In Table 10, we present the statistical analysis of the mining results. In Table 10, we list the comparisons among the proposed algorithm and the other three algorithms in terms of maximum *L*_*k*_ size and number of rules. According to the data in the table, the proposed algorithm outperforms the other three algorithms in mining.

**Figure 8:**
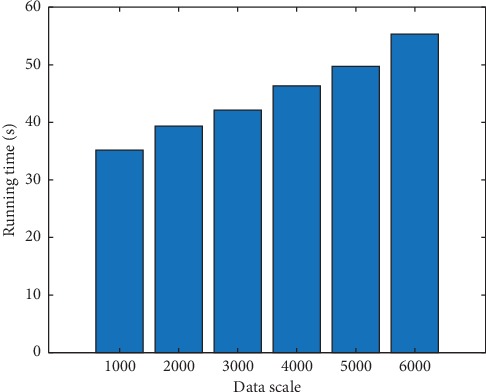
The running time of algorithm in a different scale of data samples.

To further evaluate the performance of the proposed algorithm in big data environment, this article further gives the running time of the algorithm in a different scale of data samples. The running time result of the algorithm is shown in Figure 8. The *min_sup* value and the *min_conf* value are 0.7 and 0.6, respectively. As can be seen in Figure 8, with the growth of data scale, although running time has increased, it is still within the acceptable range. Therefore, the proposed algorithm has a very broad application prospect for big data environment.

Considering the advantages of parallel computing in big data environment, the proposed algorithm can further improve the running speed through parallel computing [40–42]. The proposed algorithm needs to scan the data repeatedly in the calculation of frequent itemsets. It can search for frequent sets in parallel on multiple data blocks by dividing the original data into blocks, and finally merge the results of parallel calculation into the frequent itemsets required by the proposed algorithm.

### 5.2. Rules Evaluation

In this subsection, we will compare the rules that were mined by two methods and explain rules that are listed in Tables 11 and 12. The analysis and explanation of the mining rules reveal that the proposed algorithm can effectively mine TARs from BF data. Furthermore, these mining rules provide an effective theoretical basis for decision-making. Because the interval of the time series is equal to 30 min and *T* *=* *6*, if *X* occurs, after 3 hours *Y* will occur. The values of min_Sup, min_*Conf*, min_CF, and min_Re*cency* are set to 0.6, 0.6, 0.4, and 40, respectively, and 69 rules can be mined by our algorithm, whereas only 6 rules can be mined by the algorithm that was proposed by Dang et al. [23].

According to Table 12, the rules that are mined by Dang et al. [23] contain only two items; hence, the algorithm proposed by Dang et al. can only find a temporal relationship between two variables in BF. However, such knowledge can be easily duplicated with expert knowledge; thus, the reference value is not high. By contrast, the rules that are mined by the proposed algorithm can discover knowledge among multiple variables, and as we discussed previously, obtaining this knowledge from expert experience is difficult. Moreover, the min_*CF* threshold and *Lift* value that are used in this article were selected for demonstrating the performance of TARs. *Recency* guarantees the timeliness of TARs that are mined by the proposed algorithm. Thus, TARs that are mined by the proposed algorithm facilitate decision-making and ensures the stable conditions of BF.

Comparing Tables 11 and 12, the algorithm that is proposed in this article can efficiently mine the rules among multiple items and discover the implicit rules. Compared with the rules obtained by the algorithm proposed by Dang et al. [23], the rules that were mined by our algorithm can describe the relationship among multiple items more clearly.

We consider *Rule*{12,43⟶92,102, *Confidence*=0.93939, *Lift*=1.1136, *Recency*=88.888, *CF*=0.61266}} as an example. The antecedent of this rule corresponds to the following: if the blast volume is fluctuating normally, but oxygen enrichment is ascending, then the blast furnace bosh gas volume and theoretical combustion temperature will fluctuate normally after 3 hours. Because the *Recency* is 88.888, this rule is not out-of-date. The *Lift* exceeds 1 and satisfies the min_*CF* threshold; hence, this rule is an effective strong association rule.

As another example, consider *Rule* : {43,52⟶92,102, *Confidence*=0.94236, *Lift*=1.1172, *Recency*=84.1761, *CF*=0.63164}. If oxygen enrichment is ascent and the top temperature is fluctuating normally, then the blast furnace bosh gas volume and the theoretical combustion temperature will fluctuate normally after 3 hours. According to the *Recency*, *Lift*, and *CF* of this rule, it is an effective strong association rule.

The variable relation in the blast furnace is highly complicated; a change in one variable may have the other effects; hence, it is difficult to predict impact of several current operations on the furnace conditions. However, the algorithm that is proposed in this article can discover association rules among multiple items. According to *Lift* and *CF*, the rules that are mined by our algorithm are effective. The experimental results demonstrate the rules that are excavated by our algorithm can provide effective evidences to facilitate decision-making.

### 5.3. Rules Updating

**Figure 9:**
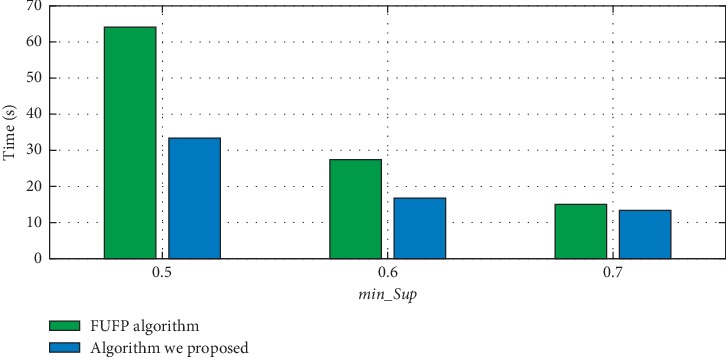
The updating speed in different min_*Sup*.

**Figure 10:**
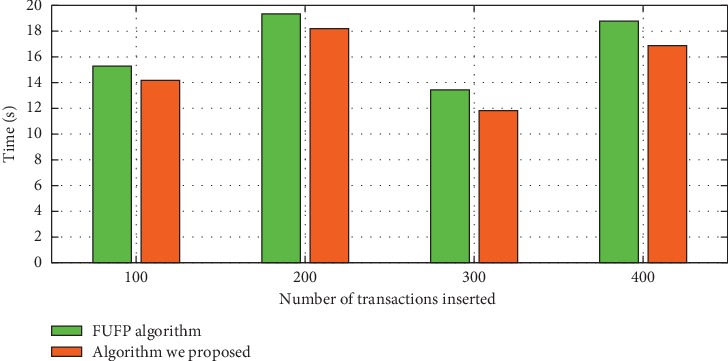
The updating speed in different number new transactions.

In this section, we will compare the rule updating speed of the Pre-FUFP algorithm and the rule updating algorithm in this article when new data are inserted. Experiment results are shown in Figures 9 and 10. First, we assume there are 1038 transactions in the original database, and 400 transactions are inserted, then we set different min_sup to evaluate the efficiency of two algorithms, and the experiment result is shown in Figure 9. Then, we still make the original database contain 1038 transactions and change the size of inserted transactions. The experiment results show that the algorithm that is proposed in this article can run better than the Pre-FUFP algorithm. Therefore, the proposed rule updating algorithm makes it possible to apply the time-series association rule mining algorithm that is proposed in this article to the dynamic database.

### 5.4. ANFIS Combined with the Proposed TAR for Predicting the Permeability Index

To further evaluate the performance of the rules that are mined by the proposed algorithm, we combine the adaptive neuro-fuzzy inference system (ANFIS) and the association rules to predict the permeability index (PI) which is an important evaluating indicator in BF.

ANFIS is an adaptive fuzzy inference system that combines the self-learning function of neural networks with the inference function of fuzzy systems. In the fuzzy inference system, the knowledge base directly affects the final prediction results. The rules that are mined by ARM have the same if-then form as the inference rules in the fuzzy system. This motivates us to apply the mined association rules to ANFIS. In this article, the fuzzy rule-base will be replaced by the association rules that are mined by the proposed algorithm and conventional TARs. Then, we will compare the results with those of conventional ANFIS to evaluate and illustrate the performance of the proposed algorithm.

According to expert experience, six variables are selected in this article: blast volume, blast temperature, blast pressure, oxygen enrichment, top temperature, and actual blast velocity. The membership functions of the input variables are Gaussian functions. We use 1240 sets of data as training data of ANFIS and the other 154 sets of data as testing data.

**Figure 11:**
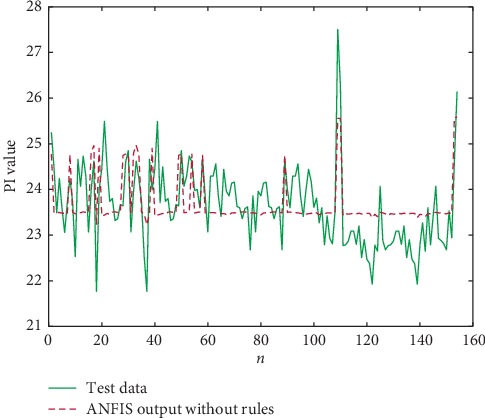
ANFIS output without TARs.

**Figure 12:**
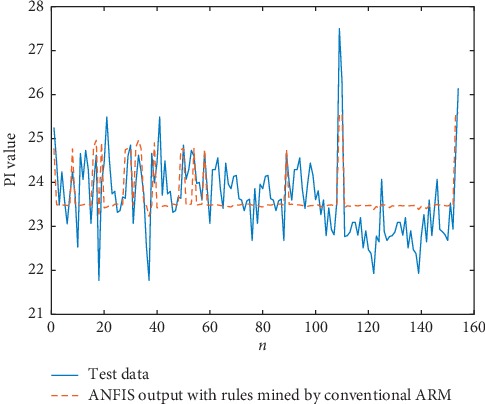
ANFIS output with rules mined by LTARM.

**Figure 13:**
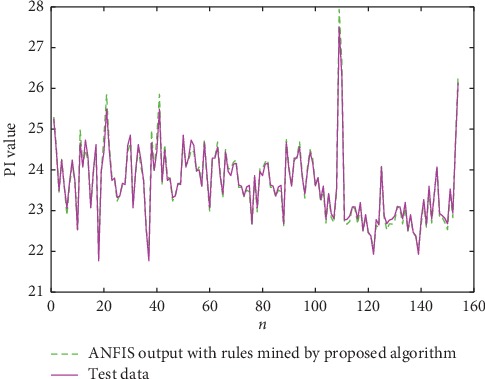
ANFIS output with rules mined by the proposed algorithm.

The combination of ANFIS and the rules that are mined by our algorithm converges at 3000th iterations, whereas the ANFIS without rules and with rules that are mined by LTARM both converge 8000 times. The predicted outputs of PI are shown in Figures 11–13.

The comparison results of RMSE are presented in Table 13. Combining the TARs that are mined by our algorithm with ANFIS can accelerate the training of the neural network, and the output of ANFIS using the TARs is more accurate than that of the original ANFIS. This experiment further demonstrates and illustrates the practicality of the rules we have excavated via our algorithms.

## 6. Conclusion

In this article, to solve the problems of mining TARs with generality and discovering implicit knowledge, a novel knowledge mining algorithm-based data are proposed. To tackle the problem of identifying outdated rules, a time decay function is proposed to avoid making wrong decisions with the out-of-date rules. Moreover, a rules updating algorithm is proposed to maintain the association rules mined in the dynamic database of BF. To ensure the validity of rules, we introduce *CF* and *Lift* to evaluate the association rules that are mined by the proposed algorithm.

Experimental results demonstrate that the proposed algorithm outperforms the traditional methods in terms of both the number of frequent itemsets and the number of rules. Furthermore, the proposed algorithm can efficiently mine the temporal relationships among multiple items, and according to the value of *Lift* and *CF*, most of the rules that are mined by the proposed algorithm are effective strong association rules. Therefore, by applying the proposed algorithm to BF production, one can discover more useful knowledge from temporal information among multiple variables, which guarantees the stable and smooth operation of BF. Furthermore, the updating algorithm that is proposed in this article can update the rules in dynamic database. In summary, the proposed algorithm is a satisfactory TARM mining algorithm in terms of the number of rules and the effectiveness of the rules. Last, we combine ANFIS and the TARM algorithm that is proposed in this article to further evaluate the performance of the algorithm, and we obtain satisfactory results by experiments.

The main contributions of this article are as follows: it proposes a novel algorithm to mine implicit TSAR from BF production data and overcomes the problem of rules coinciding with expert experience, which is encountered by traditional methods that are used in BF. A time decay function is used to identify the out-of-date rules. In addition, a rule-updating algorithm is proposed for maintaining association rules in dynamic databases. However, several problems remain to be solved. First, we will attempt to extract rules more efficiently. Second, a more efficient mining algorithm will be adopted to accelerate the mining process. Third, we will apply our algorithm to other fields to solve more complex problems.

## Figures and Tables

**Algorithm 1:**
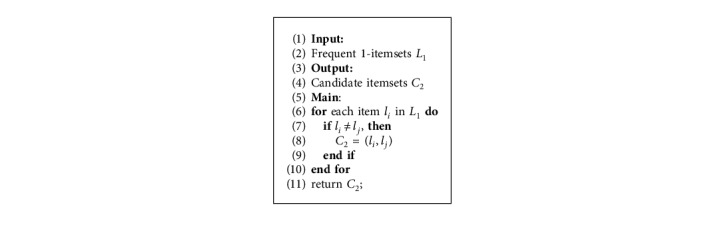
Generating candidate itemsets *C*_2_.

**Algorithm 2:**
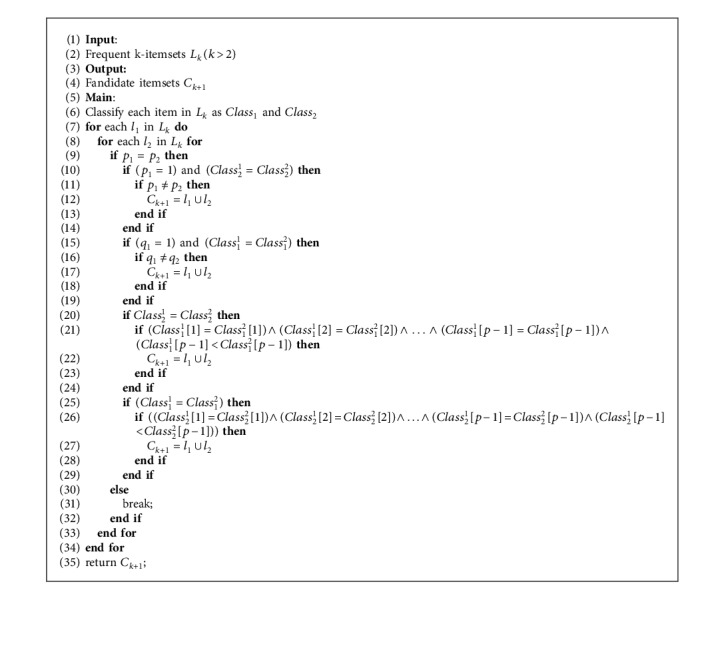
Generating candidate itemsets *C*_*k*+1_.

**Algorithm 3:**
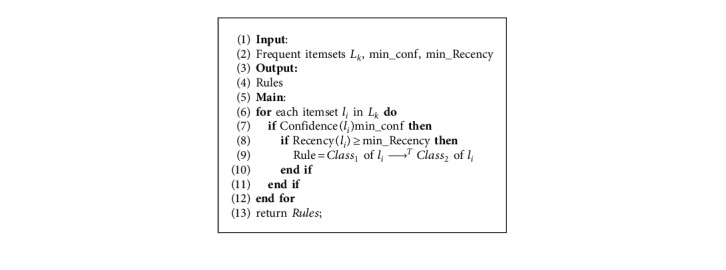
Rules generation.

**Algorithm 4:**
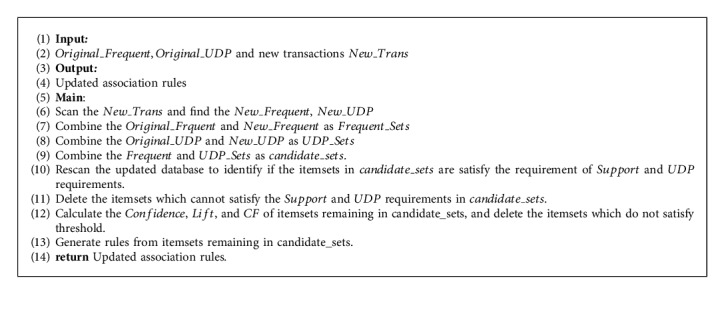
Rules updating.

**Table 1:** The log database used in this example.

Transaction ID	Transaction time	Items
1	2018/11/1 0:00	*a*, *c*, *d*
2	2018/11/1 0:05	*a*, *c*, *d*
3	2018/11/1 0:10	*a*, *d*
4	2018/11/1 0:15	*a*, *e*
5	2018/11/1 0:20	*a*, *c*, *e*
6	2018/11/1 0:25	*a*, *e*
7	2018/11/1 0:30	*f*
8	2018/11/1 0:35	*b*, *c*, *e*
9	2018/11/1 0:40	*b*, *d*, *e*, *f*
10	2018/11/1 0:45	*a, c*

**Table 2:** The result of *Timelist*(*i*) and *Count*(*i*) of each item in *D*.

Item	*Timelist*	*Count*
*a*	1, 2, 3, 4, 5, 6, 10	7
*B*	8, 9	2
*C*	1, 2, 5, 8, 10	5
*D*	1, 2, 3	3
*E*	4, 5, 6, 8, 9	5
*F*	7, 9	2

**Table 3:** The result of *Timelist*(*i*) and *Support* of each item in *D*.

Item	*Timelist*	*Support*
*A*	1, 2, 3, 4, 5, 6, 10	0.7
*B*	8, 9	0.2
*C*	1, 2, 5, 8, 10	0.5
*D*	1, 2, 3	0.3
*E*	4, 5, 6, 8, 9	0.5
*F*	7, 9	0.2

**Table 4:** The result of *Timelist*(*i*) and *Count*(*i*) of candidate 2 − *itemsets*.

*Itemsets*	*Count*	*Timelist*
(*a*⟶*b*)	2	{5, 6}
(*a*⟶*c*)	2	{2, 5}
(*a*⟶*e*)	5	{1, 2, 3, 5, 6}
(*a*⟶*f*)	2	{4, 6}
(*b*⟶*a*)	0	Null
(*b*⟶*c*)	0	Null
(*b*⟶*e*)	0	Null
(*b*⟶*f*)	0	Null
(*c*⟶*a*)	2	{1, 2}
(*c*⟶*b*)	1	{5}
(*c*⟶*e*)	3	{1, 2, 5}
(*c*⟶*f*)	0	Null
(*e*⟶*a*)	0	Null
(*e*⟶*b*)	1	{5}
(*e*⟶*c*)	1	{5}
(*e*⟶*f*)	1	{4}
(*f*⟶*a*)	1	{7}
(*f*⟶*b*)	0	Null
(*f*⟶*c*)	1	{7}
(*f*⟶*e*)	0	Null

**Table 5:** Nine cases and their results.

*Case: new-original*	Results
Case 1: frequent-frequent	Always frequent
Case 2: frequent-UDP-Sets	Rescan database, but at least is UDP-sets
Case 3: frequent-small	Rescan database
Case 4: UDP-sets-frequent	Rescan database, but at least is UDP-sets
Case 5: UDP-sets-UDP-Sets	Rescan database, but at least is UDP-sets
Case 6: UDP-sets-small	Rescan database, but at least is UDP-sets
Case 7: small-frequent	Rescan database
Case 8: small-UDP-sets	Rescan database
Case 9: small-small	Always small

**Table 6:** Input variables and their corresponding discretization intervals.

Input	Descent	Normal fluctuation	Ascent
Blast volume	<3400	3400 ~ 3500	≥3500
Blast temperature	<1170	1170 ~ 1190	≥1190
Blast pressure	<335	335 ~ 350	≥350
Oxygen enrichment	<4400	4400 ~ 5000	≥5000
Top temperature	<100	100 ~ 140	≥140
Normal blast velocity	<190	190 ~ 200	≥200
Actual blast velocity	<220	220 ~ 230	≥230
Permeability index (PI)	<23	23 ~ 26	≥26
Blast furnace bosh gas volume	<4400	4400 ~ 4500	≥4500
Theoretical combustion temperature	<2200	2200 ~ 2300	≥2300
Permeability coefficient	<6	6 ~ 7	≥7

**Table 7:** Interval division and coding.

Interval division	Descent	Normal fluctuation	Ascent
Coding	1	2	3

**Table 8:** Variable coding.

Input	Encoding number
Blast volume	1
Blast temperature	2
Blast pressure	3
Oxygen enrichment	4
Top temperature	5
Normal blast velocity	6
Actual blast velocity	7
Permeability index (PI)	8
Blast furnace bosh gas volume	9
Theoretical combustion temperature	10
Permeability coefficient	11

**Table 9:** Rules number mined by the proposed algorithm with different *T*.

*T*	Rules number
(*T*=1)	85
(*T*=2)	82
(*T*=3)	64
(*T*=4)	73
(*T*=5)	72
(*T*=6)	69
(*T*=7)	68
(*T*=8)	79
(*T*=9)	78
(*T*=10)	80

**Table 10:** Comparisons of mining results of four algorithms.

min_conf	min_sup	*L*_*k*_				Rules number			
		Proposed algorithm	Dang et al.	Khen and Simon	FP tree	Proposed algorithm	Dang et al.	Khen and Simon	FP tree
0.6	0.6	6	2	1	2	86	11	0	16
	0.7	5	2	1	2	36	6	0	6
	0.8	3	1	1	1	3	0	0	0

0.7	0.6	6	2	1	2	82	11	0	15
	0.7	5	2	1	2	35	6	0	6
	0.8	3	1	1	1	3	0	0	0

0.8	0.6	6	2	1	2	80	9	0	13
	0.7	5	2	1	2	35	6	0	6
	0.8	3	1	1	1	3	0	0	0

**Table 11:** Rules example mined from blast furnace data with the proposed algorithm.

Rules	*Confidence*	*Lift*	*Recency*	Certain factor
12,43⟶102	0.9394	1.0562	88.8880	0.4519
12,52⟶102	0.9612	1.0807	77.3956	0.6492
12,63⟶92	0.9969	1.1359	77.9292	0.9744
12,63⟶102	0.9593	1.0786	77.9292	0.6322
52,63⟶102	0.9608	1.0802	72.8792	0.6453
43,52⟶92	0.9827	1.1198	84.1761	0.8587
43,52⟶102	0.9597	1.0790	84.1761	0.6351
43,52⟶92,102	0.9424	1.1172	84.1761	0.6316
52,63⟶92	0.9897	1.1277	72.8792	0.9157
52,63⟶102	0.9608	1.0802	72.8792	0.6453
63⟶92,102	0.9167	1.0868	85.6909	0.4679
12,43⟶92,102	0.9394	1.1136	88.8880	0.6127

**Table 12:** Rules example mined from blast furnace data with TSARM.

Rules	*Confidence*	*Lift*	*Recency*	Certain factor
12⟶92	0.9505	1.0686	90.4124	0.5522
12⟶102	0.9612	1.0807	77.3956	0.6492
32⟶92	0.9367	1.0633	60.7867	0.4827
32⟶102	0.9457	1.0633	60.7867	0.5092
52⟶102	0.9365	1.0530	85.6652	0.4260
63⟶92	0.9504	1.0829	85.6909	0.5946

**Table 13:** The comparison results of RMSE.

Method	RMSE
ANFIS output without TARs	0.0447
ANFIS with rules mined by LTARM	0.0443
ANFIS with rules mined by the proposed method	0.0143

## Data Availability

The stock data used in this article have already given the open access address in the text, but because of the confidentiality agreement, the blast data cannot be disclosed.
